# Establishment of a novel protocol for assessing the severity of subarachnoid hemorrhage in circle Willis perforation mouse model

**DOI:** 10.1038/s41598-024-60237-w

**Published:** 2024-05-02

**Authors:** Rui Zhang, Dilaware Khan, Sajjad Muhammad

**Affiliations:** 1 Department of Neurosurgery, Medical Faculty & University Hospital Düsseldorf, Heinrich Heine University Dusseldorf, Mooren Str. 5, 40225 Dusseldorf, Germany; 2 Department of Neurosurgery, University of Helsinki and Helsinki University Hospital, Helsinki, Finland

**Keywords:** Circle Willis perforation mouse model, Neurological assessment, ROB score, Subarachnoid hemorrhage, Neuroscience, Diseases, Neurology

## Abstract

The Circle of Willis perforation (cWp) mouse model is a key tool in subarachnoid hemorrhage (SAH) research; however, inconsistent bleeding volumes can challenge experimental reliability. To address this issue, we introduced the ROB Scoring System, a novel protocol integrating Rotarod Tests (RT), Open-field Tests (OT) video analysis, and daily Body Weight Loss (BWL) monitoring to precisely categorize SAH severity. Forty *C57BL/6* mice underwent cWp SAH induction, categorized by ROB into severity subgroups (severe, moderate, mild). Validation compared ROB trends in subgroups, and ROB outcomes with autopsy results on postoperative days three and seven for acute and sub-acute evaluations. Mortality rates were analyzed via the survival log-rank test, revealing a significant difference among SAH subgroups (*P* < 0.05). Strong correlations between ROB grades and autopsy findings underscored its precision. Notably, the severe group exhibited 100% mortality within 4 days post SAH onset. Single parameters (RT, OT, BWL) were insufficient for distinguishing SAH severity levels. The ROB score represents a significant advancement, offering an objective method for precise categorization and addressing inherent bleeding variations in the cWp SAH model. This standardized protocol enhances the reliability and effectiveness of the SAH translational research, providing a valuable tool for future investigations into this critical area.

## Introduction

There is no optimal animal model to mimic the clinical picture of subarachnoid hemorrhage (SAH) making the research in the field of SAH more complex and challenging. The circle of Willis perforation (cWp) model and the cisterna magna injection model are the two often used mouse models for preclinical SAH research. Both models have their own pitfalls that have been previously reviewed^1,2^. The cWp SAH mouse model is a widely utilized approach to investigate the pathophysiological outcomes of SAH during the early phase and to explore early brain injury (EBI) directed pharmacological targets^3,4^. The severity of SAH can however affect the neurological outcome very similar to the human situation. The better grade SAH with less Fischer bleeding score generally has better outcomes.

A consistency in volumes of blood in subarachnoid space is important to yield comparable disease severity to include these animals for pharmacological interventions. However, it is challenging to precisely control bleeding volume during cWp SAH induction, leading to significant post-operative variability in severity among individual mice^2^. The inherent limitation of the cWp SAH mouse model poses difficulties in standardizing experimental conditions, especially in SAH treatment testing studies, which distinguishes it from the injection model of SAH^5^. This variability presents a significant challenge when attempting to directly compare the effectiveness of specific treatments between groups, potentially introducing bias into the evaluation process.

To address this challenge and establish a consistent starting point for preclinical SAH treatment research using the cWp SAH mouse model, it is essential to implement a neurological scoring system for assessing disorder severity post SAH induction. While various methods have been proposed for assessing post SAH status, not all are tailored to SAH treatment research or offer objective parameters^6,7^. Existing protocols for assessing SAH severity often rely on third-party observers who are blinded to the animals' status. However, these protocols present challenges due to the subjective criteria employed by different observers, leading to potential bias in assessment. In contrast, our ROB scoring system incorporates three easily monitored and evaluated parameters: video-analysis open-field test, Rotarod test, and daily body weight loss. Each parameter is designed to be objective and can be repeated consistently by different observers. Firstly, we utilize the mouse's ear as a monitor target in the video-analysis open-field test. Even subtle movements such as scratching or sniffing can cause ear displacement, which is accurately detected by the camera. This method ensures sensitivity surpassing that of human observation. For example, in our practice, even the severe SAH mouse in a “frozen” status can be monitored through an ear displacement by the camera. Secondly, the Rotarod test records the duration of the animal's running on a rotating bar. When the animal drops from the bar, the duration time is automatically recorded, providing an objective measure of motor function. Lastly, daily body weight loss serves as a sensitive indicator of the rodent's health status post-SAH. Additionally, it reflects the animal's willingness and ability to access food and water. Each item is assigned a different score. Based on the test results, the scores of all items are added up to get the final test score. The severity of the bleeding is then distinguished based on the total score (Table 1). By integrating these objective parameters, the ROB scoring system offers a robust and precise means of assessing post-SAH status, minimizing bias and enhancing the reliability of SAH preclinical research. The ROB score enhances objectivity and precision in assessing post-SAH status in live animals. The aim here is to improve the quality of translational studies focused on SAH research.

**Table 1:** The criteria of ROB score.

Score	Rotarod test (s)	Open-field test (distance)	Body weight loss
1	0–50	0–300	> 20%
2	51–100	300–500	15–20%
3	101–200	500–800	10–15%
4	201–299	800–1000	5–10%
5	300	> 1000	< 5%

Each mouse underwent daily evaluations, during which it received a performance score ranging from 1 to 5 based on its performance in individual tests. The cumulative scores from all tests were then aggregated to derive a final ROB score. Subarachnoid hemorrhage severity criteria of ROB score: severe (3–6), moderate (7–10), and mild (11–15).

Besides, Sugawara et al.^8^ introduced a grading system for assessing the severity of a rodent model. They utilized high-resolution images of the brain ventral surface, specifically focusing on the Circle of Willis and basilar arteries. In these images, the basal cistern was divided into six segments. Each segment was allotted a grade from 0 to 3 depending on the amount of subarachnoid blood clot in the segment as follows: Grade 0: no subarachnoid blood, 1: minimal subarachnoid blood, 2: moderate blood clot with recognizable arteries, 3: blood clot obliterating all arteries within the segment (Supplementary Table S1, Supplementary Fig. S1). The autopsy score method carried both significant advantages and disadvantages. While it is widely recognized that autopsy remains the most accurate means to assess SAH status following perforation, its inherent disadvantage lies in the irreversible nature of the procedure. Once mice are euthanized to obtain brain samples, there is no opportunity for further evaluation, rendering it unsuitable for long-term dynamic neurological assessments. In this study, the autopsy score will serve as a criterion to evaluate the accuracy of the ROB score. Randomly selected mice will be euthanized on postoperative days three and seven, and their brain samples will be assessed according to the autopsy grading system. then the results will be compared with the ROB score. To ensure the objectivity of the ROB score by validating it against the autopsy findings at different time points during the postoperative period (Supplementary Figs. S2, S3, Supplementary Tables S2 and S3).

## Results

### Mice population, ROB score evaluation, and group classification

**Figure 1:**
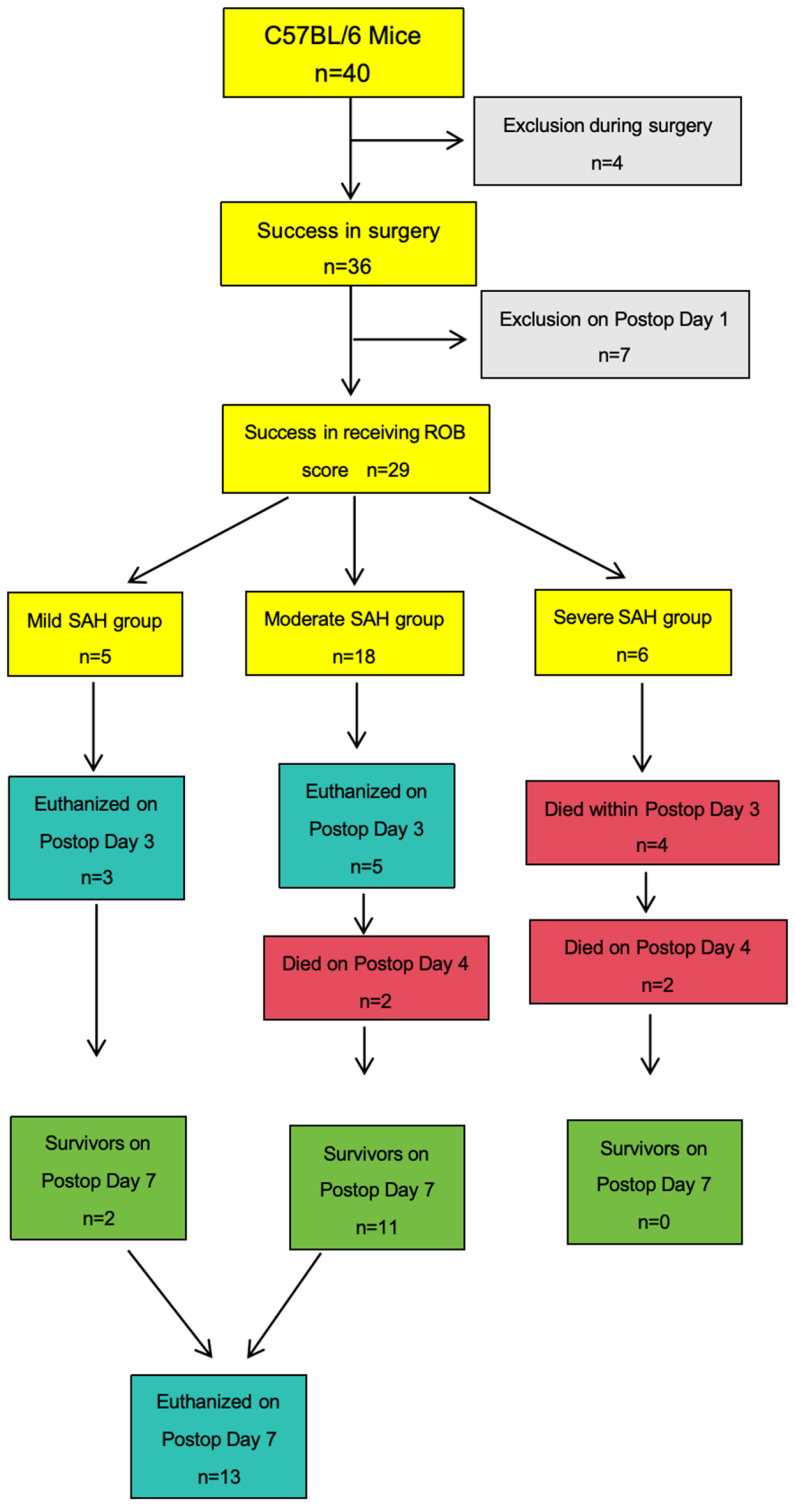
SAH group workflow of The ROB score experiment. SAH group workflow of ROB experiment. Exclusion were made intra- and post- operative. Eight mice were randomly chosen and euthanized on post SAH day three. The survivors on post SAH day seven were all euthanized.

From the original cohort of 40 SAH group mice, exclusions were made as follows: one mouse was removed from the study due to an unexpected neck tumor, one mouse died during the surgical procedure due to the rupture of ICA, two mice the filament insertion was not optimal. These exclusions resulted in a total of 36 mice considered valid for the research. Subsequently, four mice were excluded due to death or euthanasia within the first 24 h that can not get their first ROB score. Three mice were excluded due to missing data for postoperative day one (RT machine fault). The remaining 29 mice, post ROB grading, exhibited a spectrum of scores, ranging from 4 to 13. This variation led to the categorization of 6 mice into the severe SAH group, 18 into the moderate SAH group, and 5 into the mild SAH group based on the ROB score on the postoperative day one (Fig. 1, Table 1).

**Figure 2:**
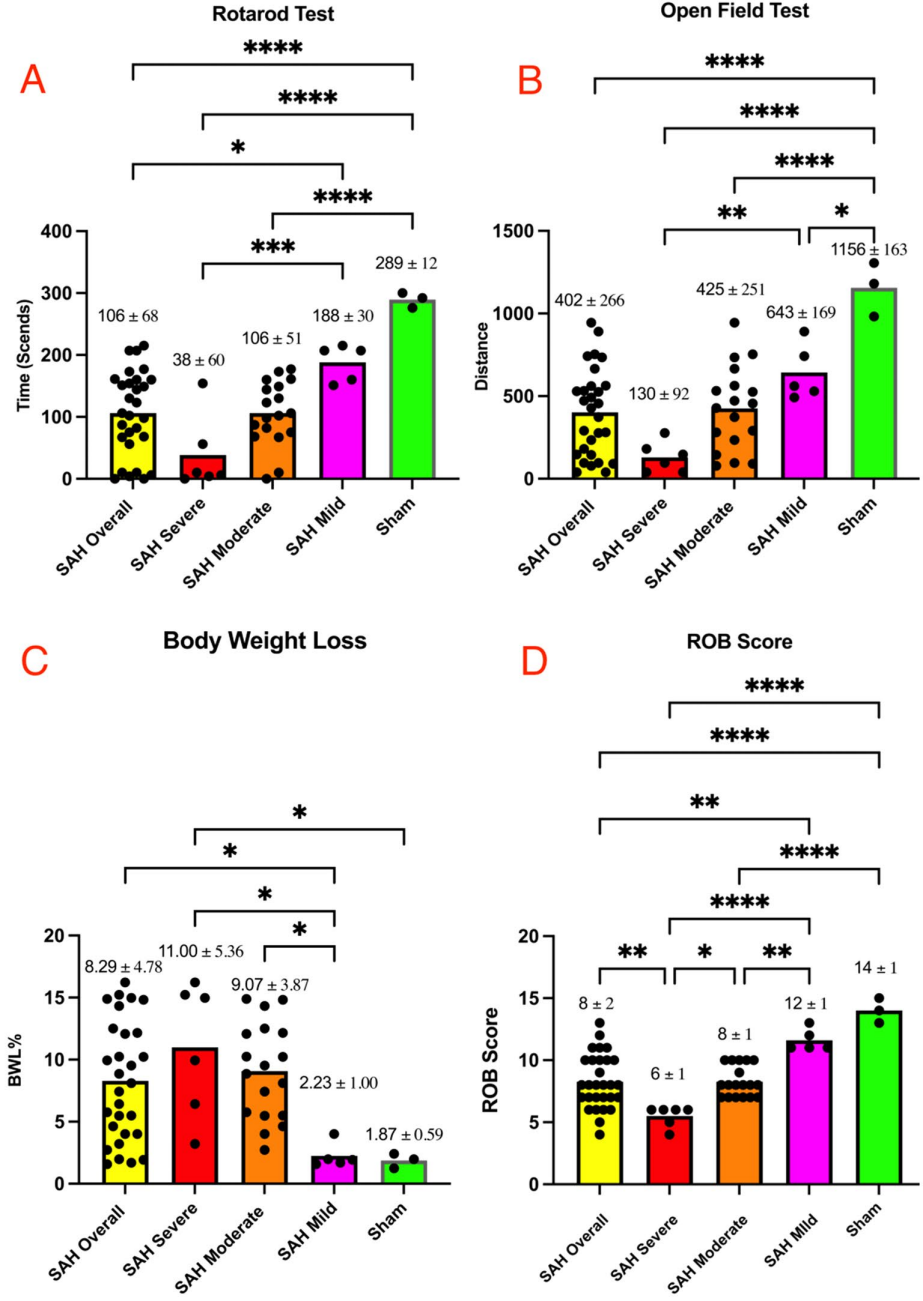
Comparative analysis of performance metrics and ROB score across experimental groups on post subarachnoid hemorrhage (SAH) day one. (**A**) Rotarod test results: ordinary one-way ANOVA multiple comparisons revealed significant differences (P < 0.05) between overall SAH and Sham outcomes. Among SAH groups, only severe and mild subgroup outcomes exhibited significant differences. (**B**) Open-field test results: significant differences (P < 0.05) were observed between Sham and SAH overall groups and within Sham vs. each SAH subgroup. However, significance was found only in the severe vs. mild subgroup comparison within the SAH subgroups. (**C**) Body weight loss: no significant differences were observed in the comparison between Sham and SAH overall groups. However, significant differences (P < 0.05) were noted within SAH subgroups, particularly in the severe vs. mild, and moderate vs. mild subgroup comparisons. (**D**) ROB score: significant differences (P < 0.05) were evident between the Sham and SAH overall groups and within SAH groups. The ROB score effectively distinguished the neurological performance of SAH mice.

Three mice from the Sham group underwent surgery and were subsequently euthanized after neurological assessment on postoperative day one. Their ROB scores ranged from 13 to 15. The data obtained from the Sham group mice served as a baseline for comparison with the SAH group mice. (Tables 1, 2, Fig. 2, Supplementary Table S4).

**Table 2:** The postoperative results of mortality, Rotarod test, open-field test, and body weight loss in each group.

Groups	Mortality(D3)	Mortality(D7)	Rotarod test (D1)		Open-field test (D1)		Body weight loss (D1)	
Overall (SAH)	13.79	38.1	Range	0–215	Range	39–944	Range	1.57–16.23
			Mean ± SD	106 ± 68	Mean ± SD	402 ± 266	Mean ± SD	8.29 ± 4.78
Severe (SAH)	66.67	100	Range	0–154	Range	39–277	Range	3.21–16.23
			Mean ± SD	38 ± 60	Mean ± SD	130 ± 92	Mean ± SD	11.00 ± 5.36
Moderate (SAH)	0	15.38	Range	0–177	Range	90–944	Range	2.73–14.89
			Mean ± SD	106 ± 51	Mean ± SD	425 ± 251	Mean ± SD	9.07 ± 3.87
Mild (SAH)	0	0	Range	151–215	Range	492–891	Range	1.57- 4.00
			Mean ± SD	188 ± 30	Mean ± SD	643 ± 169	Mean ± SD	2.23 ± 1.00
Sham	NA	NA	Range	276 -300	Range	982 -1305	Range	1.24—2.40
			Mean ± SD	289 ± 12	Mean ± SD	1156 ± 163	Mean ± SD	1.87 ± 0.59

Unit: mortality and body weight loss: percentage (%). Rotarod test: time (seconds). Open-field test: distance (absolute value without unit). Mice in SAH groups: overall n = 29; Severe n = 6; Moderate n = 18; Mild n = 5. Mice in Sham group: n = 3. *D* post-SAH day, *SAH* Subarachnoid hemorrhage, *NA* not applicable.

### Mortality

**Figure 3:**
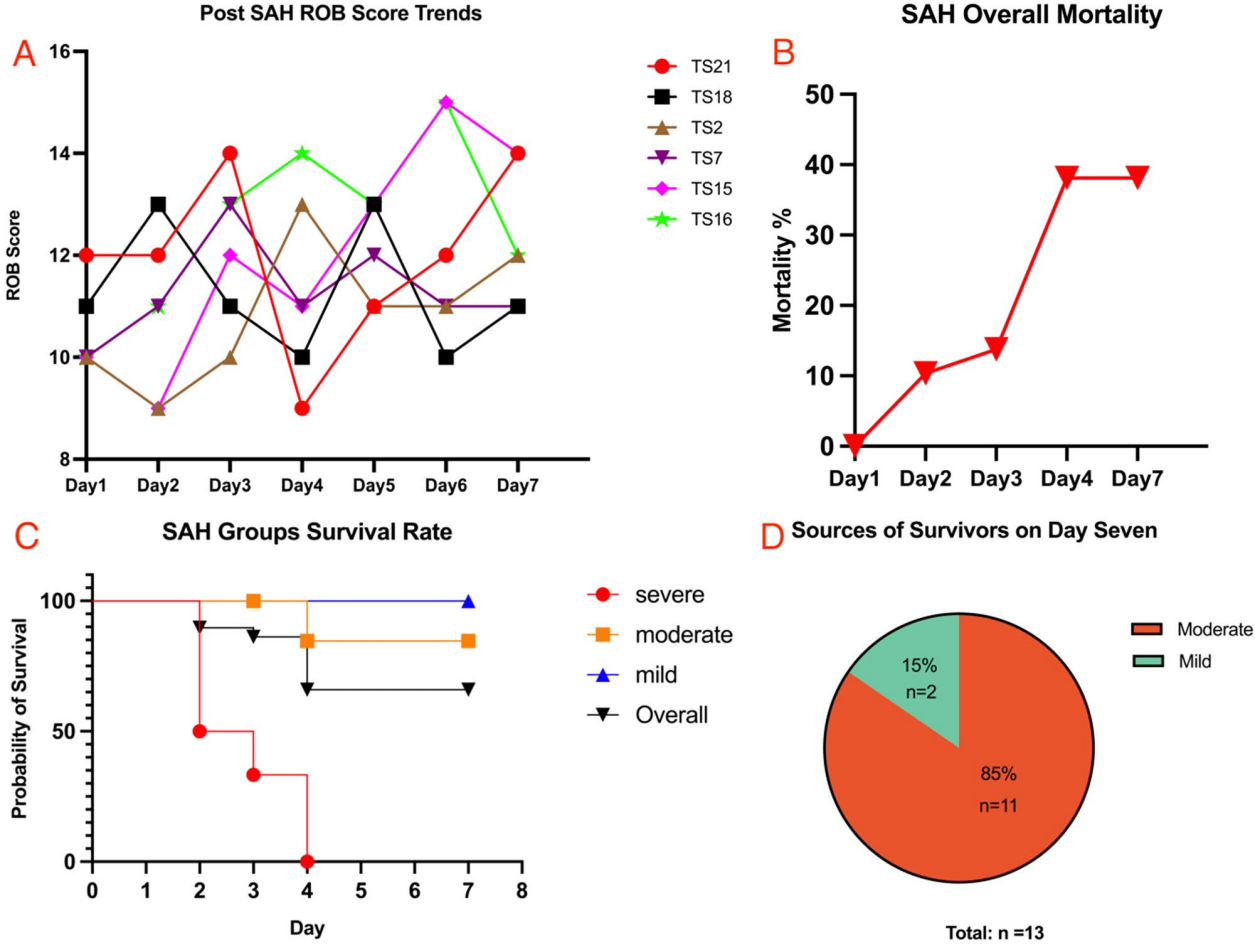
Comparative analysis of the survival subarachnoid hemorrhage (SAH) mice ROB score trends, mortality over time, survival rate in SAH subgroups and the survivors sources. (**A**) ROB score trends: retrospective analysis of the ROB scores of six randomly selected mice indicated a decreasing ROB score trend on post-SAH day four or five. (**B**) Mortality trends: the mortality of post-SAH mice increased over time. Notably, on post-SAH day four, the mortality rate reached a peak, which correlated with the decrease in the ROB score. (**C**) Survival rate comparison: survivor log-rank analysis revealed a significant difference (P < 0.05) in survival rates among the SAH subgroups. (**D**) Post-operative day seven survivor sources: thirteen mice survived on postoperative day seven, with two from the mild group and eleven from the moderate group. There were no survivors in the severe group.

The mice in the SAH group triggered the human ending point and then euthanized, were also taken into mortality calculation. The intraoperative and first 24-h mortality in the SAH induction group was 13.51% (5 of 37 mice), which is slightly lower than reported in our previous meta-review paper^2^. The overall mortality rates of the valid 29 mice at different time points were as follows: 0% (0 of 29) on post SAH day one, 10.34% (3 of 29) on day two, 13.79% (4 of 29) on day three, 38.1% (8 of 21) on day four, and 38.1% (8 of 21) on day seven. Notably, the severe group exhibited an overall mortality rate of 66.67% (4 of 6) on day three and 100% (6 of 6) on day four. In contrast, the moderate and mild groups experienced no mortality on day three. On postoperative day seven, the mortality rate for the moderate group was 15.38% (2 of 13, 1 mouse died and the other one euthanized on postoperative day four), while the mild group continued to show no death. The final survivors came from moderate and mild groups, with no mice from the severe group. Thus mortality comparison made and analyzed by survival log-rank test showed a significant difference between those groups, *P* < 0.05 (Table 2, Fig. 3). The mice from the Sham group (n = 3) had no death during the surgery until euthanasia after the neurological assessment on postoperative day one.

### Autopsy results on day three and day seven

Eight mice were euthanized on day three of the experiment. Among the randomly selected five mice, four received similar severity-level evaluations in both score systems. The autopsy examinations exhibited close alignment with the assigned ROB grades on post-SAH day three. On day seven, four mice were randomly selected from a total of thirteen. In three out of the four mice, similar scores were observed in both autopsy and ROB assessments, providing affirmation of the correlation between the assigned ROB score and autopsy examinations (Supplementary Figs. S1, S2, S3, Supplementary Tables S1, S2, S3).

### Video analysis open field test, body weight loss, and Rotarod test

We systematically recorded and conducted a comparative analysis of individual mice performance in the test within Sham and SAH subgroups on the Post SAH day one, and assigned a ROB score to the mice.

The result of RT in SAH groups (unit: seconds): Overall (n = 29): Range = 0–215, Mean ± SD = 106 ± 68; Severe (n = 6): Range = 0–154, Mean ± SD = 38 ± 60; Moderate (n = 18): Range = 0–177, Mean ± SD = 106 ± 51; Mild (n = 5): Range = 151–215, Mean ± SD = 188 ± 30. The result of RT in Sham group: Range = 276–300, Mean ± SD = 289 ± 12 (n = 3).

The result of OT in SAH groups (distance, absolute value without unit): Overall (n = 29): Range = 39–944, Mean ± SD = 402 ± 266; Severe (n = 6): Range = 39–277, Mean ± SD = 130 ± 92; Moderate (n = 18): Range = 90–944, Mean ± SD = 425 ± 251; Mild (n = 5): Range = 492–891, Mean ± SD = 643 ± 169. The result of OT in Sham group: Range = 982–1305, Mean ± SD = 1156 ± 163 (n = 3) (Supplementary Fig. S4).

The result of BWL in SAH groups (Percentage %): Overall (n = 29): Range = 1.57–16.23, Mean ± SD = 8.29 ± 4.78; Severe (n = 6): Range = 3.21–16.23, Mean ± SD = 11.00 ± 5.36; Moderate (n = 18): Range = 2.73–14.89, Mean ± SD = 9.07 ± 3.87; Mild (n = 5): Range = 1.57–4.00, Mean ± SD = 2.23 ± 1.00. The result of BWL in Sham group: Range = 1.24–2.40, Mean ± SD = 1.87 ± 0.59 (n = 3). The obtained results were meticulously documented in the accompanying table (Table 2, Supplementary Table S4).

## Discussion

The rodent models provide an important tool to investigate disease pathology, find molecular mechanisms behind and find novel drug targets^9^. Most of the animal studies in the field of stroke and SAH have failed in clinical trials^10,11^. Hence, preclinical research in the field of stroke and SAH needs methodological improvements.

Subarachnoid hemorrhage is a devastating disease and clinical outcomes heavily depend on its onset bleeding volume or its initial severity. In preclinical SAH research, the cWp SAH model or the cisterna Magana blood injection model provides excellent tools for investigating the mechanism or testing some potential drugs. However, with few strengths and weaknesses of both models that need to be addressed during preclinical pharmacological interventional studies^1,2^.

The cisterna Magna blood injection model generates a consistent blood volume in the subarachnoid space via controlled injection of particular blood volume but the model does not mimic the clinical SAH pouring blood from arterial/aneurysm rupture. The cWp SAH model on the other hand provides more resemblance with clinical SAH pouring arterial blood in the subarachnoid space with heterogeneity in blood volume producing different grades of severity very similar to the clinical situation. For investigations focusing on pathophysiological mechanisms of disease, particularly mechanisms in the EBI phase, the cWp SAH model is the most suitable currently available model. However, for preclinical pharmacological interventions using the cWp model, the uneven severity distribution may add bias. Hence, to address this aspect, the application of ROB score provides a valuable tool to unify the severity of the cWp SAH model and offers a platform for the development of potential therapeutic interventions^12,13^.

Evaluating the neurological deficits in SAH mice is a fundamental aspect of experimental design. Various protocols have been established to assess post SAH neurological outcomes. However, some of these protocols rely on subjective assessments by observers, introducing potential bias and inter-observer variability. Subjective evaluation can be influenced by individual interpretation and experience, which may impact the reliability of the results^6^. Moreover, certain assessment protocols necessitate the euthanasia of animals and subsequent autopsy to define the severity of neurological deficits. While such approaches may yield valuable data, they are inherently invasive and limit the ability to monitor long-term recovery^8,14^.

Certainly, much like in human medicine, computer tomography (CT) and magnetic resonance imaging (MRI) are powerful tools for investigating the severity of SAH in mouse models. However, it's important to acknowledge that these imaging modalities come with certain practical limitations. The equipment itself can be costly, and conducting CT or MRI checks in mouse models may not always be convenient due to various factors, including availability, logistics, and the small size of the animals^7,15,16^. Therefore, it becomes imperative for researchers to consider and develop more objective, non-invasive assessment methods that minimize observer bias and support dynamic monitoring for living animals.

In the clinical context, grading systems like the Hunt and Hess Scale^17^ or Glasgow Coma Scale (GCS)^18^ have long served as valuable tools to evaluate and categorize the severity of SAH in human patients. Inspired by Hunt & Hess scale and GCS, we introduced a novel grading system known as the ROB score.

On post-SAH day one, the neurological tests indicated a significant difference (*P* < 0.05) in performance between the overall SAH group and the mice in the sham group, with the SAH group showing lower performance. Interestingly, when comparing the SAH subgroups with the sham group based on ROB scores, it was observed that the SAH mild group exhibited no significant difference compared to the sham group. This finding mirrors observations in clinical practice, where human patients with low Hunt–Hess grades typically remain asymptomatic or experience only slight headaches. Two important revelations emerge from these findings. Firstly, intraoperative intracranial pressure monitoring is essential to confirm the success of SAH induction. Without this confirmation, neurological assessments alone cannot distinguish between mild SAH mice and sham-operated mice. Secondly, comparing two SAH groups with unequal numbers of mild SAH mice is meaningless, especially in treatment testing. The group with more mild-level SAH mice would inevitably yield better outcomes, leading to comparisons inaccurate (Fig. 2).

In the subsequent days, the tests were repeated daily with mice from the SAH groups, and the ROB score was updated accordingly. Upon retrospective analysis of the ROB scores of the survivors, an intriguing pattern emerged. Among the randomly chosen six mice, four exhibited a decrease in ROB score on post-SAH day four, while the remaining two mice displayed this pattern on day five. Concurrently with the fluctuation in ROB scores, mortality rates increased, reaching a peak on post-SAH day four. This observation is similar to the progression of human SAH and underscores the value of the ROB score in assessing SAH severity (Fig. 3).

When comparing the results of the ROB categorization with the autopsy grading results, satisfactory alignment was observed. On postoperative day three, four out of five randomly selected samples exhibited concordant results with the autopsy findings. Similarly, on postoperative day seven, three out of four randomly chosen samples demonstrated congruent outcomes. It is noteworthy that this observed bias is within an acceptable range both in the acute and sub-acute phases of SAH, however, in the chronic phase, the ROB score will be more accurate than the autopsy score due to the resorption of the SAH (Supplementary Figs. S2, S3, Supplementary Tables S2, S3).

Indeed, our study employing the ROB score to categorize the severity of SAH in mouse models revealed an another interesting observation. The mortality in subgroups demonstrated an obvious bias, however, the post-operative status of these mice couldn't be accurately distinguished by any single parameter (RT, OT, and BWL). For instance, when considering BWL, there was no significant difference between the severe and moderate groups (11.00 ± 5.36% vs. 9.07 ± 3.87%, *P* = 0.7877). A similar lack of significance was also observed in RT and OT. However, it's crucial to note that postoperative mortality exhibited substantial deviations (*P* < 0.05) among these groups, with stark differences observed on post SAH day three and day seven. There was also a bias in the source of survivors, in total thirteen mice survived on the post-SAH day seven, and no mouse from the SAH severe group. That means no single parameter can adequately discern the severity of the SAH mouse. However, when these parameters are amalgamated using the ROB score, a more precise identification of the severity of the SAH mouse becomes feasible. This observation also emphasizes the enhanced value of the ROB score in delivering a thorough and nuanced assessment (Fig. 3).

The introduction of the ROB system represents a significant advancement in the field of SAH research to accurately allocate mice with different disease severity in control and interventional groups providing accurate data on the efficacy of tested drugs. Here are a few aspects of our SAH severity score that will certainly enhance the quality of preclinical SAH research. (1) Precise Classification of SAH Severity: The ROB grading system excels in its ability to precisely classify SAH models into three distinct subgroups: severe, moderate, and mild. This refined categorization of SAH severity is essential to our study as it enables us to differentiate the data within each subgroup. For instance, on postoperative day three, the overall mortality rate stands at 13.79%. However, within the severe group, this rate soars to 66.67%, representing nearly fivefold higher mortality. This striking disparity highlights a fundamental insight—a simplistic division of experimental subjects into "Sham" and "SAH" groups falls short of capturing the complexity of the condition. A more detailed classification is imperative. In practice, dichotomous categorization of "Sham" and "SAH" would disregard the inherent diversity in SAH severity, potentially leading to misleading or inconclusive findings, especially in the drug treatment trial. So this precision in classification results in a more comprehensive understanding of SAH and its outcomes, ultimately elevating the quality and relevance of our research. (2) Dynamic Evaluation and Timely Identification: The ROB grading system not only provides a static assessment of SAH severity but also offers dynamic evaluation. The ability to assess and update the ROB grade daily after the surgery is invaluable. This dynamic assessment aids in the prediction of adverse outcomes, including the death of mice. By recognizing the ROB score as a predictive tool for identifying severe cases, our research contributes to the advancement of animal welfare. This early identification and intervention can mitigate suffering and improve the ethical treatment of research animals. (3) Promoting Uniformity in Experimental Models: By offering an accurate and refined method for identifying the severity of SAH, the ROB score serves as a innovative tool in making the cWp SAH model closely resemble the injection model in the SAH severity uniform aspect. This innovation represents a substantial stride towards providing a standardized experimental SAH mouse model for various applications. The practical implications of this uniformity are profound. In drug treatment studies, for instance, exclusion of severe or mild SAH mouse models could be possible, the uniformity of the cWp SAH model ensures that experimental outcomes are less influenced by variations in disease severity. This reliability in experimental conditions enhances the validity of findings and strengthens the foundation for potential therapeutic interventions.

Now, the cWp SAH model plus ROB score could meet these demands: (1) reproducible and consistent blood deposition in the subarachnoid space. (2) uniformity, controlled degree of hemorrhage. (3) mechanism of hemorrhage closely simulating aneurysmal SAH. (4) blood distribution correlating with aneurysmal SAH. (5) ease to performance and (6) reasonable costs^19^.

However, being a novel protocol, it is essential to acknowledge the inherent limitations of our study. The sample size in our experiment is relatively small, and we recognize the necessity for the SAH translational research community to adopt and further validate our protocol in their investigations. As the sample size increases in future studies, it may be prudent to consider adjustments to the criteria of the ROB for enhanced robustness and generalizability.

## Materials and methods

### Animal population and ethical statement

In our study, a cohort of 4-month-old male *C57BL/6* mice wild type (n = 40) with a body weight of around 25.3–35.5 g were employed to establish this ROB Scoring System for assessing SAH severity (Janvier Laboratory Le Genest-Saint-Isle, France) according to the guidelines [§ 8 Abs. 1. The Animal Protection Act (TierSchG) and § 31. Animal Welfare Experimental Animal Regulations (TierSchVersV)] of the Animal Care Committee of the District Government of North Rhine-Westphalia (Protocol Number: 81-02.04.2021.A195), meanwhile, additional three male *C57BL/6* mice wild type (n = 3) with body weight from 25.2 g to 33.3 g were utilized as Sham control. We confirmed all of the surgery and experiments according to the ARRIVE guidelines, and they were approved by the Animal Care Committee of the District Government of North Rhine-Westphalia.

### Criteria for ROB scoring system and post SAH neurological assessment

The methodology incorporated three objective parameters: RT, video analysis OT, and daily BWL measurements. Each parameter was scored on a scale ranging from 1 to 5, contributing to a comprehensive final score within the range of 3–15. The ROB methodologically categorizes the post SAH mice into three subgroups based on the disorder severity: severe (3–6), moderate(7–10), and mild (11–15) (Table 1).

Neurological assessments were conducted daily following SAH induction. On post-SAH day one, the performance in RT, OT, and BWL of mice from both Sham and SAH groups was recorded and compared. According to the ROB score criteria, all mice were assigned a ROB score, after which the SAH group was further subdivided into three subgroups. SAH groups continued assessing until day seven, and the Sham group mice were euthanized on day one after ROB scoring (Table 2, Supplementary Table S4).

### Subarachnoid hemorrhage cWp mouse model:

According to the previous research^4,20,21^, the SAH induction procedure was described in brief: General anesthesia was administered using isoflurane, with an initial induction of 5% isoflurane in combination with 2 L per minute of oxygen, followed by maintenance at 1.75–2.5% isoflurane^22^. After exposing the left common carotid artery (CCA), external carotid artery (ECA), and internal carotid artery (ICA), a 5–0 monofilament (5–0 Prolene Ethicon) was carefully introduced into the ICA, passing through the CCA bifurcation. The suture was then further advanced into the ICA until resistance was encountered, typically at a distance of approximately 15–18 mm from the CCA bifurcation. A further advancement was applied to ensure perforation of the wall at the bifurcation of the anterior and middle cerebral arteries. An intracranial pressure monitor was applied to confirm the success of the perforation^23^. Subsequently, the suture was retracted, and the ICA was re-perfused. The surgical incision was meticulously closed in a standard manner. The mice from the Sham group underwent the same procedure without filament perforation. Following the surgical procedure, the mice were allowed to recover and were individually housed until the scheduled time of euthanasia. Throughout the study, all animals were provided free access to food and water with the human control day-night rhythm.

### Video-monitoring open field test

The post SAH mice underwent behavioral assessments in an open-field apparatus, a cubic polyvinyl chloride box measuring 42 × 42 × 42 cm^24^. A 10-s video clip was recorded using a fixed-cell phone camera with parameters set to 30 frames per second (FPS). Subsequently, these video clips were subjected to analysis using the open-source software "Tracker" (version 6.1) available at https://physlets.org/tracker/.

The procedure involved several sequential steps. Initially, the mouse was placed within the test field and gently immobilized at the center of the arena by securely holding its tail. Video recording was initiated, and subsequently, the mouse was released to observe its spontaneous reactions and motion. The recorded video was imported into the Tracker software. The analysis was commenced by identifying the initial video frame where the mouse resumed its unrestricted movement. Subsequently, a specific video segment was designated for analysis, encompassing the following 160 frames, which spanned a 5-s interval. The analysis key frames were strategically positioned at five-frame intervals. The movement tracking analysis was initiated, utilizing one of the mouse's ears (either the left or right) as a tracking marker. The position of the selected ear was assessed in each key frame at the five-frame intervals.

The Tracker software automatically computed the data, including the mouse's movement trajectory and distances covered over the 5-s duration. This data was then exported and saved in Excel files. To facilitate further comparison, the OT results were recorded by using the absolute value of the moving distance. The whole process will be completed within 2 min by a skilled investigator.

### Rotarod test and body weight measurement

Each day, prior to conducting the Rotarod test, the body weight of the mice was measured and recorded with a precision of 0.1 g. The formula of BWL was (body weight^preoperative^ − body weight ^postoperative^) $\div$ body weight^preoperative^ $\times$ 100%.

The RT apparatus was configured in an acceleration mode, with the rotational speed ranging from 4.0 to 40 RPM (Rotation Per Minute). Subsequently, the mice were carefully positioned on the rotating rod and subjected to testing, continuing until the animal either fell from the rod to the base or the designated endpoint of 300 s, as stipulated by our experimental protocol^25^. This testing procedure was repeated three times for each mouse, and the results were duly recorded. The final analysis was based on the average performance data from these three trials.

### Autopsy score

The randomly chosen mice were euthanized by cardiac perfusion under deep anesthesia. The brains of euthanized mice were harvested and subsequently examined. Any subarachnoid haemorrhage or blood clot present on the vertical surface of the brain samples was then assigned a score according to the autopsy criteria^8^ (Supplementary Fig. S1, Supplementary Table S1). On post-SAH day three, a subgroup of eight mice was randomly selected for euthanasia. Brain samples from these mice were evaluated. This evaluation entailed a comparative analysis between the ROB score and the autopsy score during the acute phase. The remaining mice were continually observed until day seven, at which point they were euthanized. Their autopsy findings were also compared with the ROB score to assess the ROB score’s precision in the sub-acute phase. This process was conducted by another researcher who was blinded to the mice’s status and ROB score. The data obtained from this assessment were recorded and compared (Supplementary Figs. S2, S3, Supplementary Tables S2, S3).

### Statistical analysis

Mortality, RT, OT, and BWL in each subgroup were statistically analyzed. Mortality was compared between subgroups with the Survival Log Rank Test, statistical significance was assumed at *P* < 0.05. To compare ROB score of Sham and SAH subgroups, the RT, OT, and BWL from subgroups, we used a one-way ANOVA to find any statistical difference, a *P* < 0.05 was considered as statistically significant. All statistical analyses were performed with R (v3.6).

### Ethics approval

The Animal Care Committee of the District Government of North Rhine-Westphalia (Protocol Number: 2021.A195).

### Supplementary Information

Supplementary Information.

## Data Availability

All data are available in the main text or the supplementary materials.

## Abbreviations

cWp: Circle of Willis perforation
SAH: Subarachnoid hemorrhage
RT: Rotarod tests
OT: Open-field tests
BWL: Body weight loss
EBI: Early brain injury
CCA: Common carotid artery
ECA: External carotid artery
ICA: Internal carotid artery
FPS: Frames per second
RPM: Rotation per minute
CT: Computer tomography
MRI: Magnetic resonance imaging
GCS: Glasgow Coma Scale
