# Supplementary material for: Establishment of a novel protocol for assessing the severity of subarachnoid hemorrhage in circle Willis perforation mouse model
Source: Sci Rep. 2024 May 2;14:10147. doi: 10.1038/s41598-024-60237-w (PMC11066000; doi:10.1038/s41598-024-60237-w)
Supplement: Supplementary file 1 — Supplementary Information. [file 41598_2024_60237_MOESM1_ESM.pdf]

Supplementary materials of "Establishment of A Novel Comprehensive protocol for Assessing the Severity of Subarachnoid Hemorrhage in Circle Willis Perforation Mouse Model"

Table S1: Autopsy Grade Criteria

Score	Criteria
0	no subarachnoid blood
1	minimal subarachnoid blood
2	moderate blood clot with recognizable arteries
3	blood clot obliterating all arteries within the segments

‡ Mild SAH: 0-7, Moderate SAH: 8 – 12, Severe SAH:13 – 18. SAH: Subarachnoid Hemorrhage.

Figure. S1: Schematic Image of Autopsy Score Criteria

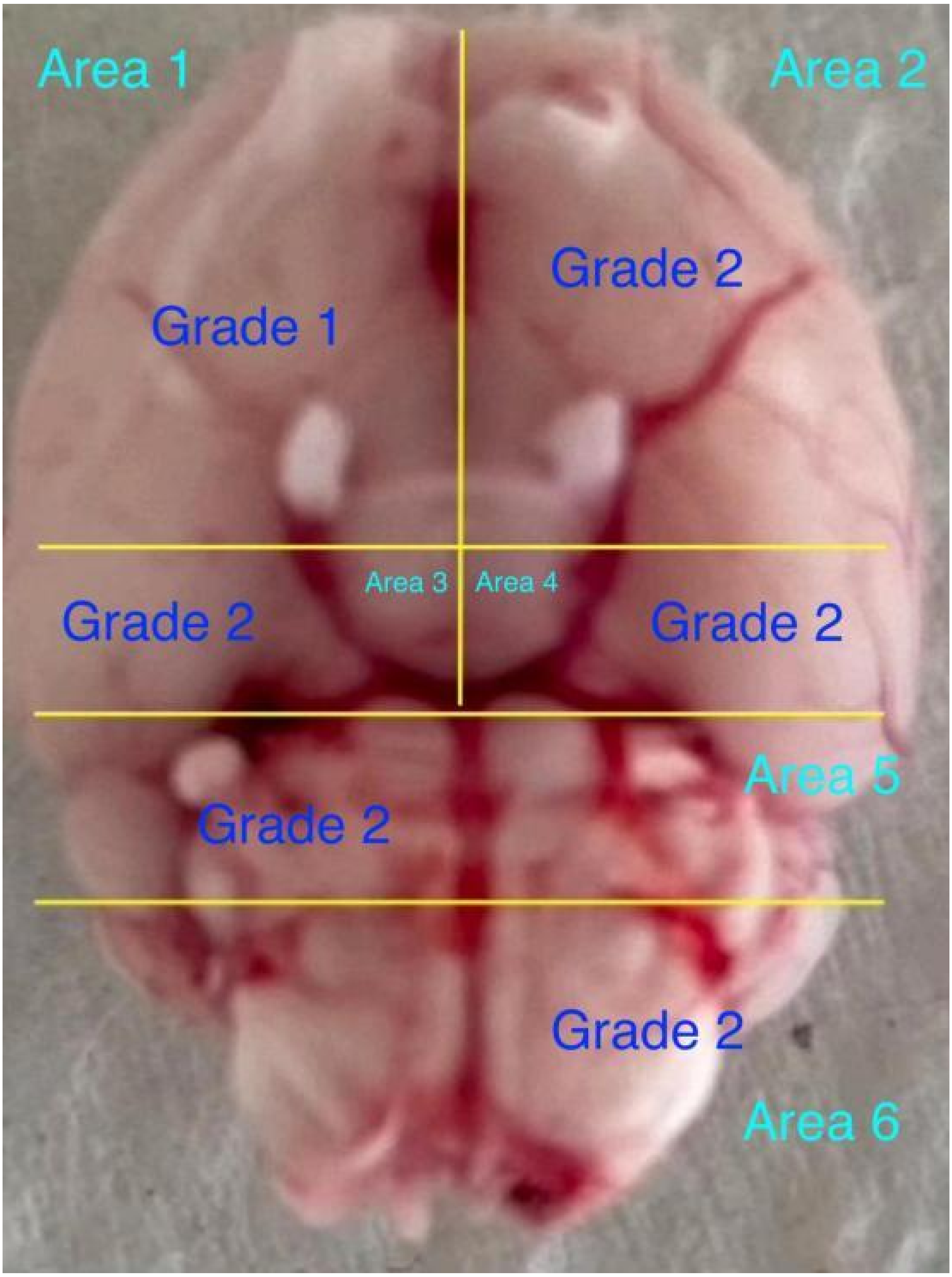

Fig. S1: The ventral surface of the mouse brain was divided into six distinct areas. Each of these areas received a score ranging from 0 to 3 based on specified criteria.

Figure. S2: Original Image of Postoperative Day Three Autopsy

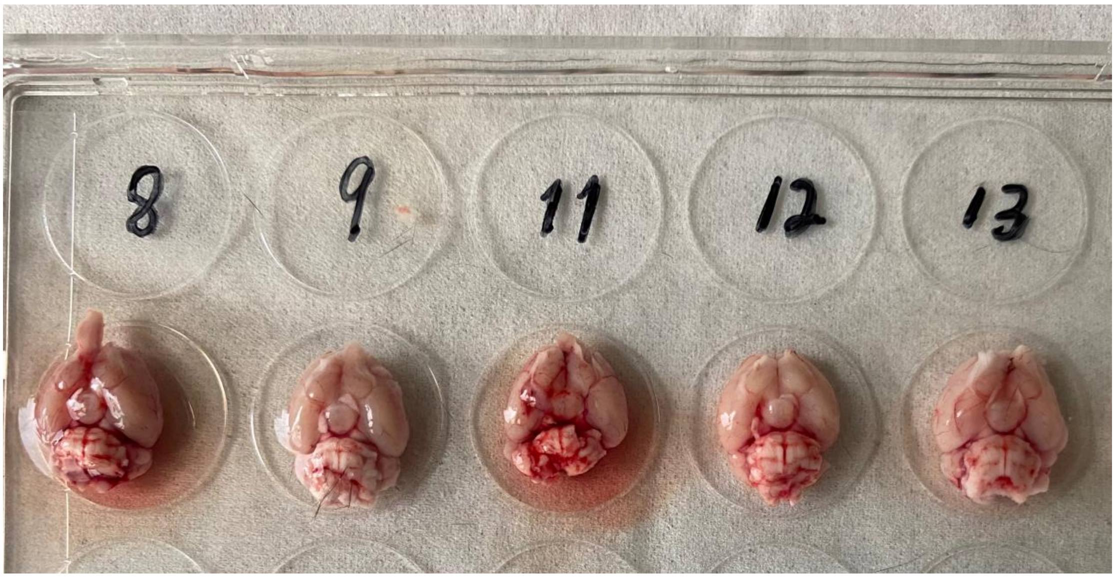

Fig. S2: Illustrates the original images of five randomly selected mice from a cohort of eight euthanized specimens. The variance observed in subarachnoid hemorrhage (SAH) levels on postoperative day three is assessed through an autopsy score.

Table S2: Post-operative Day Three Comparison Between Autopsy Score and ROB score

Animal ID	T8	T9	T11	T12	T13
Autopsy Area 1	2	1	2	0	1
Autopsy Area 2	1	0	1	0	1
Autopsy Area 3	2	2	3	2	1
Autopsy Area 4	2	2	3	2	2
Autopsy Area 5	2	1	2	2	2
Autopsy Area 6	2	1	2	1	2
Autopsy score	11	7	13	7	9
ROB score	10	14	8	11	10
Autopsy severity	moderate	mild	severe	mild	moderate
ROB severity	moderate	mild	moderate	mild	moderate

‡Autopsy on the postoperative day three revealed the high correlational signs between the autopsy score and ROB score

Figure. S3: Original Image of Postoperative Day Seven Autopsy

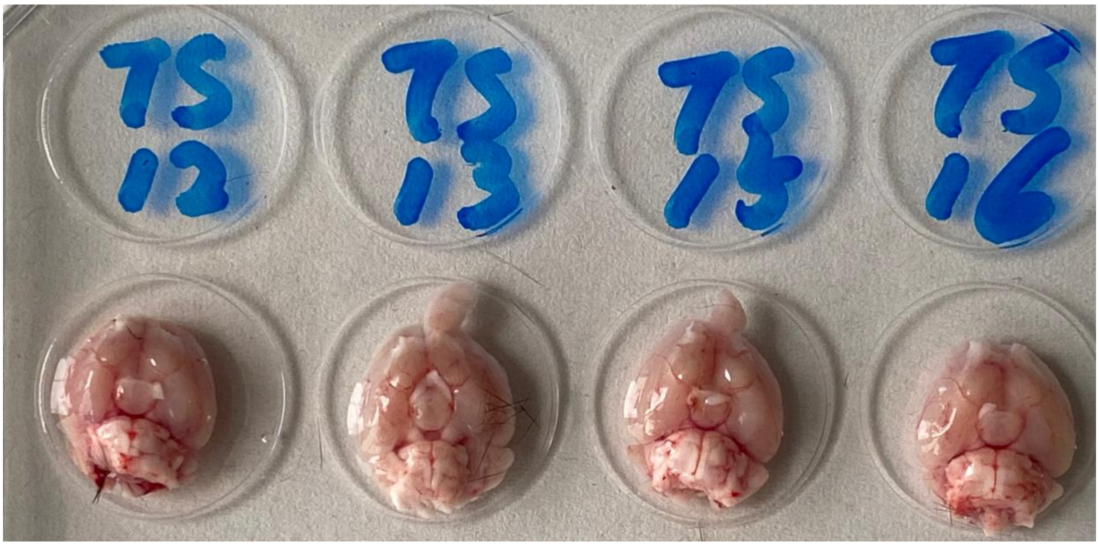

Fig. S3: Original images indicated variance in subarachnoid hemorrhage (SAH) level on the postoperative day seven, evaluated by the autopsy score.

Table S3: Postoperative Day Seven Comparison Between Autopsy Score and ROB Score

Animal ID	TS12	TS13	TS15	TS16
Autopsy Area 1	0	1	0	0
Autopsy Area 2	0	0	0	0
Autopsy Area 3	2	0	2	2
Autopsy Area 4	2	0	2	2
Autopsy Area 5	1	1	1	1
Autopsy Area 6	1	0	0	1
Autopsy Score	6	2	5	6
ROB score	12	8	14	12
Autopsy severity	mild	mild	mild	mild
ROB severity	mild	moderate	mild	mild

‡Autopsy on the postoperative day seven revealed the high correlational signs between the autopsy score and ROB score.

Figure. S4: Trajectory Analysis of Mouse Movement in The Open-field Test

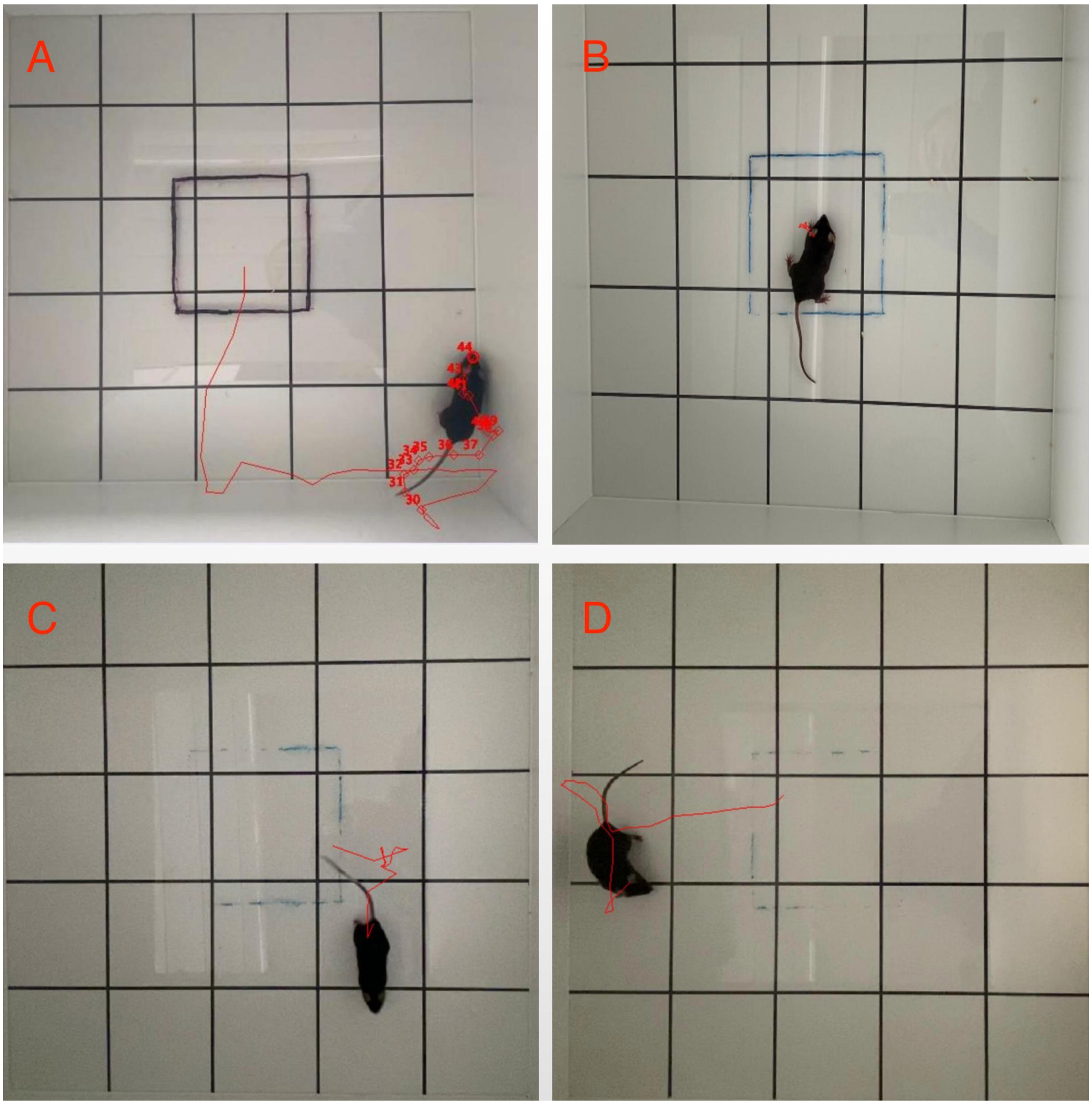

Fig. S4: The video analysis software "Tracker" was utilized to automatically analyze and generate trajectories illustrating the movement patterns of mice in the arena. The red line represents the tracked movement distances for mice in different conditions.

A: Depicts a sham mouse exhibiting swift and positive movement during testing.

B: Illustrates a mouse in a severe condition, displaying minimal movement and almost frozen throughout the testing process.

C: Illustrates a moderate mouse movement, covering approximately half the distance compared to the sham mouse.

D: Shows a mild status mouse movement, which closely resembles the movement pattern of the sham mouse.

Table S4: Daily ROB Score Records and Outcomes for Individual Mouse from Groups

ID	Group	D1	D2	D3	D4	D5	D6	D7	Outcome
T12	Mild	13	10	11					Euthanized D3
TS21	Mild	12	12	14	9	11	12	14	Euthanized D7
T9	Mild	11	14	14					Euthanized D3
T15	Mild	11	12	14					Euthanized D3
TS18	Mild	11	13	11	10	13	10	11	Euthanized D7
T11	Moderate	10	10	8					Euthanized D3
TS2	Moderate	10	9	10	13	11	11	12	Euthanized D7
TS7	Moderate	10	11	13	11	12	11	11	Euthanized D7
TS15	Moderate	10	9	12	11	13	15	14	Euthanized D7
TS16	Moderate	10	11	13	14	13	15	12	Euthanized D7
TS12	Moderate	9	7	11	10	9	14	12	Euthanized D7
T16	Moderate	8	8	6					Euthanized D3
TS4	Moderate	8	9	11	12	14	13	10	Euthanized D7
TS8	Moderate	8	5	6	5	7	6	8	Euthanized D7
TS10	Moderate	8	10	11	10	10	11	9	Euthanized D7
TS13	Moderate	8	4	5	9	8	10	8	Euthanized D7
TS19	Moderate	8	10	10	11	13	11	14	Euthanized D7
T4	Moderate	7	X	7					Euthanized D3
T8	Moderate	7	10	10					Euthanized D3
T13	Moderate	7	8	10					Euthanized D3
TS1	Moderate	7	5	4	0	0	0	0	Died D4
TS17	Moderate	7	5	3	3	0	0	0	Euthanized D4
TS20	Moderate	7	7	8	8	7	9	9	Euthanized D7
T3	Severe	6	X	0					Died D3
T17	Severe	6	4						Euthanized D2
TS5	Severe	6	4	0	0	0	0	0	Died D3
TS9	Severe	6	6	4	0	0	0	0	Died D4
TS11	Severe	5	3	3	0	0	0	0	Died D4
TS14	Severe	4	0	0	0	0	0	0	Died D2
F18	Sham	14							Euthanized D1
F19	Sham	13							Euthanized D1
F32	Sham	15							Euthanized D1

‡ D: Postoperative Day. X: Missed data due to the Rotarod machine failure.
